# Effect of spices formulations on the physicochemical and sensory properties of *Nnam gon*, a Cameroonian dish prepared with cucurbitaceae seeds

**DOI:** 10.1002/fsn3.447

**Published:** 2016-11-23

**Authors:** Josiane E. Manejo Djiogue, Richard M. Nguimbou, Leopold N. Tatsadjieu, Yannick D. Mang, Nicolas Y. Njintang

**Affiliations:** ^1^ Department of Food Science and NutritionENSAIUniversity of NgaoundereNgaoundereCameroon; ^2^ University Institute of TechnologyUniversity of NgaoundereNgaoundereCameroon; ^3^ Department of AgricultureLivestock and By productsHigher Institute of SahelUniversity of MarouaMarouaCameroon; ^4^ Department of Biological SciencesFaculty of ScienceUniversity of NgaoundereNgaoundereCameroon

**Keywords:** cucurbitaceae cake, physicochemical characteristics, quantitative descriptive analysis, spices

## Abstract

*Nnam gon*, a cake made by steam cooking a mixture of Cucurbitaceae seeds paste and others ingredients specially spices, is a highly prized dish in Central African region. A preliminary investigation conducted as part of this study highlighted that formulations used in the processing of *Nnam gon* vary according to the spices used. This study was carried out to determine the best formulation for the preparation of this dish. For this purpose, *Nnam gon* samples were produced from four formulations which differ according to the number of spices used: F0 (no spices); F1 (0.91 g of *Allium cepa* paste); F2 (0.88 g of *A. cepa* paste, 0.35 g of *Allium sativum* paste, 0.41 g of fresh *Officinale zingiber* paste, 0.41 g of fresh *Petroselinum crispum* paste, 0.33 g of *Monodora myristica*, 0.48 g of fresh *Celery graveolens* paste, 1.19 g of fresh *Allium porrum* paste, 0.13 g of *Allium lepidophyllus* powder, and 0.13 g of *Piper nigrum*), and F3 (0.90 g of *A. cepa* paste, 0.35 g of *A. sativum* paste, and 0.42 g of fresh *O. zingiber* paste). The samples were evaluated for their physicochemical characteristics and sensory profile (Quantitative Descriptive Analysis). The results revealed that proteins (16.56–17.38%), carbohydrates (4.71–5.10%), lipids (23.14–24.25%), ash (4.03–5.92%), and fibers (2.17–2.68%) increased significantly (*p* < .05) with spices adding. The increase in polyphenols (310.55–592.80 mg/100 g FM) and phytates (2.23–12.49 mg/100 g FM) contents was positively correlated with antioxidant properties of *Nnam gon* which also increased with spices adding. Significant differences were observed between the samples for all attributes generated (appearance, odor, taste, flavor, texture, and oral texture). Spices adding induced a decrease in hardness, cohesivity, elasticity, and granulous of cake but enhanced oily. *Nnam gon* produced with spicy formulation (F2 and F3) had higher mean score for general acceptance which was highly correlated (*p* < .05) with spice odor (*r* = .99), spice taste (*r* = .92), and color (*r* = .84). From this study, it is suggested that spicy Cucurbitaceae paste could improve nutritional value, antioxidant properties, and general acceptance of *Nnam gon*.

## 1 Introduction

Cucurbitaceae is an important family of plants containing about 120 genera and 800 species which are mainly distributed in tropical and subtropical regions, although a few of them are grown in temperate regions also (Teppner, 2004). The Cucurbitaceae seeds are a rich natural source of proteins and phytosterols (Fokou et al., 2009; Gemrot, Barouh, Vieu, Pioch, & Montet, 2006; Loukou et al., 2007). Evaluation of the nutritional value of Cucurbitaceae seeds revealed that it contains 42.67–56.67% crude oil, 24.79–36.21% crude protein, 2.30–17.14% crude fiber, and 1.13–4.33% ash (Badifu, 2001; Loukou et al., 2007; Azhari, Xu, Jiang, & Xia, 2014). The seeds were also found to have considerable amounts of Cu, Fe, P, Zn, K, Ca, and Mg which are essential minerals (Azhari et al., 2014).

Despite this high nutritional potential, the cultivation and use of cucurbits seeds remain limited in food industry. In fact, the world production of these seeds was estimated at 184 million tons per year (FAO, 2014), but in Cameroon the annual production is around 146,000 tons (FAO, 2010). Cameroon production is still very low compared to other countries such as South Africa (378.776 tons), Egypt (690.000 tons), and China (5.767.700 tons) (FAO, 2014). An average cost of kilogram of dry Cucurbitaceae seeds is 2.29 euros in Cameroon, and that price is one and half times the average price of kilogram of cocoa and half times that of coffee which are the main industrial crop in that country (Minader, 2012). Then, the sector of cucurbit seeds could be an important activity for agriculture in Cameroon. One way to boost the underutilized seeds production could be to increase demand for these seeds by using them as raw materials in industry or by industrializing the production of foods made from these seeds (Ebert, 2014). In this respect, improving the production of Cucurbitaceae cake could be a solution to stimulate the production of cucurbit seeds.

In fact, Cucurbitaceae cake, called *Nnam gon* in Cameroon, is a highly prized traditional dish formulated with Cucurbitaceae paste mixed with water and other ingredients such as salt, fresh eggs, oil, fish, or meat. Spices (*Allium cepa*,* Allium sativum*,* Officinale zingiber, Petroselinum crispum, Monodora myristica, Celery graveolens*, etc.) are also used to season the paste. The seasoned paste is packed in the leaves of katemfe (*Thaumatococcus daniellii*) and steam cooked (Ponka et al., 2005). This cake is prepared in all regions of Cameroon and has an important value in traditional societies of this country. It is usually offered at ceremonies like wedding and funeral (Ponka et al., 2005). Moreover, *Nnam gon* is a street food whose production is mainly ensured by women, but its consumption in the street is often linked to some digestive disorders that could probably be due to poor conditions of cooking and storage. Few studies have already been done on Cucurbitaceae cake, nevertheless the study of the nutritional value of this food realized by Ponka et al. (2005) noted that it contains around 8.96% of proteins, 13.5% of lipids, 1.86 of fibers, 1.77% of ash, and minerals such as Magnesium (108.9 mg/100 g DW), Iron (2.99 mg/100 g DW), and Zinc (3.29 mg/100 g DW).

The valorization of Cucurbitaceae seeds by industrial production of *Nnam gon* requires improving the production process, packaging, and storage conditions. In this respect, the preinvestigation conducted as part of this study has identified that the ingredients used for the formulation of *Nnam gon* vary according to household in Cameroon. Thus, it would be interesting to determine the formulation mostly accepted by the consumers. For that, chemical composition, sensory characteristics, and general acceptability of different formulated cake must be studied. For this purpose, the Quantitative Descriptive Analysis (QDA) methodology which is one of the most used descriptive approaches is recommended for sensory analysis of *Nnam gon*.

As part of main study aimed on improving the quality of this dish, this study was carried out to determine the chemical composition of *Nnam gon* produced with different formulations, their sensory characteristics, and overall acceptability.

## 2 Materials and methods

### 2.1 Sampling of Cucurbitaceae seeds

Dried samples of Cucurbitaceae seeds (*Cucumeropsis mannii*) harvested on February 2015 were purchased on March 2015 from local market in Ngaoundere, Cameroon. They were sorted manually to remove foreign matter and immature and damaged seeds. Then, the seeds were soaked for 5 min in order to facilitate dehulling which was manual. The dehulled seeds were milled using an electric grinder. Then, the flour obtained was directly used for production of *Nnam gon* samples.

### 2.2 Production of *Nnam gon* samples

**Figure 1:**
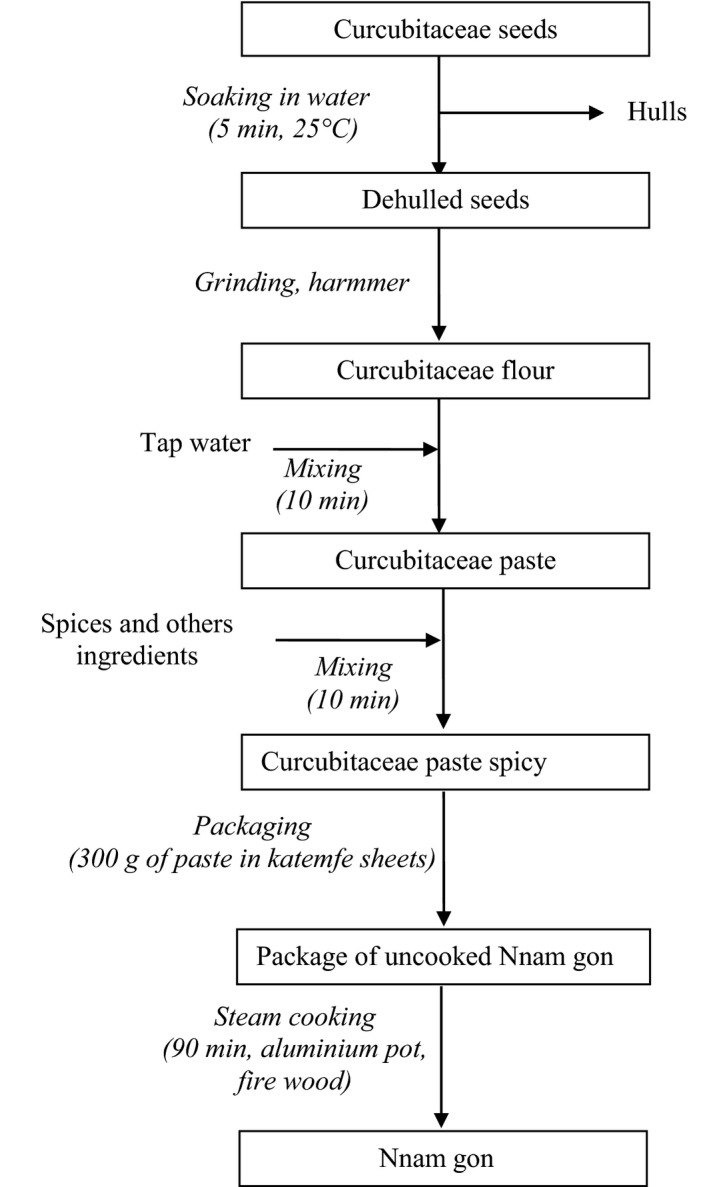
Diagram of production of Cucurbitaceae cake called *Nnam gon*

The Cucurbitaceae seeds flour was divided into four subsamples corresponding to the different formulations (F0, F1, F2, and F3). The formulations (Table 1) and *Nnam gon* production process (Figure 1) are the results of a preliminary investigation prior to this study. As reported in Figure 1, flour and water were introduced in a large plastic bowl and mixed with a wooden spatula during 10 min. Then, salt, spices, and other ingredients were added to the mixture which was remixed for 5 min. About 300 g of the final mixture was packaged in cleaned leaves of katemfe (*Thaumatococcus daniellii*) which are traditionally used for the packaging of *Nnam gon*. Packaged samples were filled into aluminum pot (10 L of capacity, with tripod) containing 1.5 L of tap water. Five samples were deposited on the tripod and were not in direct contact with boiling water. Cooking was carried out for 90 min on hearth of three stones fed with wood. The samples of the different formula were cooked separately. At the end of cooking, the samples were removed from the pot and cooled on the bench. The cakes were separately processed into three subsamples. One subsample was used for chemical analysis, another subsample for textural analysis, and the last was used for sensorial analysis.

**Table 1:** Composition of formulated pastes (100 g) used for preparation of *Nnam gon* samples

Ingredients (g)	Formula 0	Formula 1	Formula 2	Formula 3
Cucurbitaceae flour	52.54	47.00	45.60	46.64
Water	45.63	41.19	40.05	40.96
Salt	1.83	1.82	1.77	1.81
Fresh eggs	–	3.37	3.27	3.35
Soya oil	–	0.86	0.84	0.86
Fresh fish (mackerel)	–	4.83	4.68	4.79
Onion paste	–	0.91	0.88	0.90
Dried shrimps	–	0.46	–	0.45
Garlic paste	–	–	0.35	0.35
Fresh ginger paste	–	–	0.41	0.42
Fresh parsley paste	–	–	0.41	–
Pèppè powder	–	–	0.33	–
Fresh celery paste	–	–	0.48	–
Fresh leek paste	–	–	1.19	–
Disc powder	–	–	0.13	–
Black pepper powder	–	–	0.13	–

Scientific names of spices: Onion: *Allium cepa;* Garlic: *Allium sativum;* Pèppè: *Monodora myristica;* Black pepper: *Piper nigrum;* Disc: *Afrostyrax lepidophyllus;* Leek: *Allium porrum;* Ginger: *Officinale Zingiber;* Celery: *Celery graveolens;* Parsley: *Petroselinum crispum*.

#### 2.2.1 Proximate analysis of *Nnam gon* samples

Moisture, crude protein, crude fat, and crude ash contents were determined according to the AOAC procedures (1990). Protein was calculated as *N* × 6.25. Total carbohydrate was determined after digestion in concentrated sulfuric acid (Dubois, Gilles, Hamilton, Rebers, & Smith, 1956).

#### 2.2.2 Analysis of bioactive and antinutritional factors

Polyphenol content was determined using a colorimetric method as described earlier by Mang et al. (2015). Tannin level in the cakes was determined by the colorimetric method of Makkar, Siddhuraju, and Becker (2003). The phytic acid content was evaluated using a colorimetric method as reported by Gao et al. (2007). The oxalate content was determined by a titrimetric method according to AOAC procedures (AOAC 1990).

#### 2.2.3 Antioxidant properties analysis

The reducing power of cakes was measured according to the method described by Oyaizu (1986). Evaluation of DPPH Free radical scavenging activity was determined following De Ancos, Sgroppo, Plaza, and Cano (2002) with some modifications. Chelating power of cakes was measured according to the method described by Decker and Welch (1990).

### 2.3 Textural analyses

For the texture evaluation, the freshly cooked samples of cakes were equilibrated at ambient temperature for 5 min and submitted to a compression test as described by Nourian, Ramaswamy, and Kushalappa (2003) using a computer interfaced universal testing machine (Lloyd Model LRX‐2500N) equipped with a 500 N load cell. One measurement was made per cake, three cakes were tested per formulation, and their average values taken to represent the mean texture value of test samples. From the generated texture profile, the hardness was obtained from the peak force of the first compression. Adhesiveness was obtained from the final force of the first compression. Viscoelasticity index was the ratio between the peak force of the first compression and the peak force of the second compression.

### 2.4 Sensory evaluation

#### 2.4.1 Preselection of panelists

Twenty panelists (10 women and 10 men) drawn from a subuniversity population were preselected for the descriptive sensory evaluation, based on their capacity to detect sensory differences in this kind of product.

#### 2.4.2 Development of sensory descriptors

The judges previously selected, after four meetings, have developed sensory descriptors used in the definitive tests. For this, Repertory Grid Kelly's Method (Moskovitz, 1983) was used. The four formulated cakes (F0, F1, F2, and F3) were evaluated using a sensory evaluation form constituted of nonstructured 20 cm scales for each sensory descriptor. From a common agreement, the judges chose reference materials to determine the extreme points of the intensity scales and to help in the identification of sensory characteristics of the samples.

#### 2.4.3 Selection and training of panel

Previously judges were trained following QDA (Stone & Sidel, 1993) during three sessions with a view to select the definitive panel. The final panelists were selected based on their ability to discriminate different samples and the repeatability of their results as reported by Bannwart, Bolini, Toledo, Kohn, and Cantanhede (2008).

#### 2.4.4 Sensory evaluation

The selected and trained judges participated in the sensory evaluation of *Nnam gon*. The cake samples were evaluated in individual booths under white light and provided with room temperature, water, and unsalted crackers. One cake from each formulation was used for sensory analysis. The products (5 g each) were presented in glass plate coded with three digit random numbers. All the products were assessed in four random repetitions.

### 2.5 Statistical analysis

All the results were carried out in triplicate determinations. Analysis of variances was used to determine the effect of formulation on the dependent variables. Duncan's Multiple Range Test was performed to classify samples at the significant level of 5%. Statgraphics 6.0 Program was applied for the statistical analysis. Principal component analysis (PCA) and Pearson analysis were applied using Minitab Statistical Software.

## 3 Results and discussion

### 3.1 Proximate composition of *Nnam gon*

Table 2 presents the proximate composition of *Nnam gon* samples. The moisture content in these cakes ranged around the average value of 46%. The lack of significant difference observed between moisture content of the different formulated cakes suggested no significant effect of spices on moisture content of *Nnam gon*. But the water content observed was lower compared to those of Ekomba (61.1%) and Ekwang (77.6%) which are others Cameroonian cakes produced with maize flour and Cocoyam flour, respectively (Ponka, Fokou, Beaucher, Piot, & Gaucheron, 2016).

**Table 2:** Effect of formulation on proximate composition of *Nnam gon*

Parameters (/100 g DM)	Formula/Samples			
	F0	F1	F2	F3
Water content (g)	49.33 ± 1.97^a^	46.67 ± 1.97^a^	44.61 ± 2.04^a^	45.70 ± 1.77^a^
Lipids (g)	23.14 ± 0.16^a^	24.05 ± 0.11^b^	24.25 ± 0.17^b^	24.16 ± 0.10^b^
Proteins (g)	16.56 ± 0.26^a^	17.22 ± 0.31^b^	17.38 ± 0.20^b^	17.26 ± 0.23^b^
Total sugar (g)	4.71 ± 0.28^a^	4.74 ± 0.26^a^	5.10 ± 0.15^b^	4.96 ± 0.11^a^
Reducing sugar (g)	0.10 ± 0.05^a^	0.12 ± 0.08^a^	0.18 ± 0.02^b^	0.15 ± 0.05^ab^
Ash (g)	4.03 ± 0.15^a^	4.58 ± 0.93^a^	5.92 ± 0.13^b^	5.62 ± 0.48^b^
Fibers (g)	2.17 ± 0.13^a^	2.24 ± 0.17^a^	2.68 ± 0.12^b^	2.56 ± 0.14^b^

Mean ± *SD* values with different letters within the same line differed significantly (*p* < .05) as determined by Duncan's multiple range test (*n* = 03).

The ash content of cakes was in the range 4.03–5.92% of DM. This is significantly higher than ash content of Akara (3.1%), a traditional bean cake prepared in Nigeria (Okeke & Eze, 2006). Although the Cucurbitaceae seeds are rich in minerals, the high ash content of *Nnam gon* is certainly due to the addition of spices. In this respect, the cakes from F0 formulation which have not received spice have a lower ash content compared to the cakes from F2 formulation which received nine spices. In this regard, previous studies have shown that all the spices used in this study contain minerals (Ogunka‐Nnoka & Jaja, 2012; Uhegbu, Iweala, & Kanu, 2011).

Lipids are the main components of *Nnam gon*. The fat content in the samples ranged from 23.14 to 24.25%. These values were higher than fat content of 2.7% in Jeqe (cake made with white flour) consumed in rural KwaZulu‐Natal, South Africa (Spearing et al., 2013). Adding spice had no effect on lipid content of these cakes, it would probably be linked to their low‐fat content. But addition of soy oil in the formulation also contributes to increase oil content of the cakes. After lipids, proteins are the third major constituents of *Nnam gon*. The highest crude protein content was recorded for the cake from F2 formulation (17.38%). Apart from control cake (F0) which had a lower crude protein (16.56%), no significant difference was observed between crude protein contents of others formulated cakes, but spices induced an increasing in crude protein of cake probably linked to their protein content. These protein contents are close to that of moinmoin (17.71%) (Olapade & Adetuyi, 2007) indicating that *Nnam gon* is a good source of proteins.

*Nnam gon* is also a good source of energy, its carbohydrate content ranged between 4.71 for F0 cake and 5.10% for F2 cake and spices seems to enhance carbohydrate content of cake in formulations F2 and F3 probably due to spice which are incorporated into these formulations but absent in F0 and F1. In this respect, carbohydrate increasing can be attributed to onion which had 76.71% of carbohydrate content, garlic (73.03%), ginger (72.84%), and pepper (67.59%) (Nwinuka, Ibeh, & Ekeke, 2005). The carbohydrate content of *Nnam gon* was lower than 15.2% reported for Koki (a steam‐cooked cowpea emulsion cake) (Ponka et al., 2016) and 34.02% found by Okeke et al. (2009) in Ayaraya Oka (steamed maize pudding with vegetable) a dish of Nigeria. However, no significant difference was observed between reducing sugar of all formulated *Nnam gon*. Like others components, dietary fiber content of cake significantly increased with addition of spices in formulation. Fiber content was in the range 2.17–2.68 g/100 g DM. The lowest dietary fibers were obtained for the control formulated cake F0 (2.17%), whereas the highest was for cake produced with formulation F2 (2.68%).

### 3.2 Antinutrient content of *Nnam gon*

Table 3 presents the antinutrient levels in the *Nnam gon* samples. The results of the control cake (F0) show that cooking did not induce total destruction of antinutrients compounds of *Nnam gon*. There was a significant difference (*p* < .05) among the samples. For phenolic compounds, cake produced with a control formulation (F0) had the lowest level (310.55 mg/100 g DM). This may be due to its lack of spices as compared to cake produced with F1 (467.01 mg/100 g DM), F2 (592.80 mg/100 g DM), and F3 (534.40 mg/100 g DM). In fact, the addition of eggs, oil, fish, onion paste, and shrimps (F1) approximately doubled phenolic content of cake. In addition to the foregoing ingredients, adding Garlic and ginger pastes (F3) also slightly increased phenolic content of *Nnam gon*. We noted that phenolic content of the cake increased with the number of spices introduced in the cake formulation. Studies have shown that spices such as disc, leek, and celery are rich in phenolic compounds (Belewu, Olatunde, & Giwa, 2009; Uhegbu et al., 2011). In this vein, the phenolic content of cakes produced with F2 formulation was significantly higher (*p* < .05) than that of others formulations, probably due to the several spices of this formulation.

**Table 3:** Effect of formulation on antinutritional factors of *Nnam gon*

Parameters (/100 g DM)	Formula/Samples			
	F0	F1	F2	F3
Phenolics compound (mg)	310.55 ± 5.62^a^	467.01 ± 9.71^b^	592.80 ± 3.96^d^	534.40 ± 4.71^c^
Oxalates (mg)	1.41 ± 0.01^a^	2.57 ± 0.01^b^	3.30 ± 0.01^d^	2.83 ± 0.01^c^
Phytates (mg)	2.23 ± 0.07^a^	12.49 ± 0.09^b^	11.04 ± 1.26^b^	11.04 ± 1.26^b^
Tannins (mg)	131.59 ± 6.26^a^	369.11 ± 11.41^b^	527.44 ± 3.24^d^	459.68 ± 11.41^c^

Mean ± *SD* values with different letters within the same line differed significantly (*p* < .05) as determined by Duncan's multiple range test (*n* = 03).

Comparatively, adding spices in formulation also induces an increase in the oxalate content of *Nnam gon* which ranged between 1.41 and 3.30 mg/100 g DM. The oxalate content also appears to be related to the number of spices introduced in the formulation, thus, F2 presented a high content of oxalate. In fact, some health problems (corrosive gastroenteritis, shock convulsive symptoms, low plasma calcium, high plasma oxalates, and renal damage) are caused by a consumption of high levels of oxalates (Kelsay, 1985). Specifically, oxalate content increasing would be linked to the addition of garlic (2.6 mg/g), ginger (0.7 mg/g), and onion (0.3 mg/g) which have high oxalate content compared with other spices according to the literature (Nwinuka et al., 2005; Belewu et al., 2009;  Oluwatoyin, 2014). Also, by forming insoluble calcium oxalate salts, oxalates limit the availability of calcium in the body, hence decreasing the utilization of the mineral by the bones and tissues. However, the level of oxalates in the samples was less than the threshold value (780 mg) reported for food by Noonan and Savage (1999).

Phytate contents were significantly higher (*p* < .05) in cakes with spices compared to control formulation (F0) and so this higher level confirmed that spices enhanced phytate content of *Nnam gon* which ranged between 2.23 and 12.49 mg/100 g DM. However, no significant difference was noted between the phytate content of cakes produced with others formulations (F1, F2, and F3) and their phytate levels are within safe limit (30–100 mg/100 g DM) (Onomi, Okazaki, & Katayama, 2004). It is important to observe that all the spices used for the formulation contained phytates: ginger (25 mg/g), garlic (23.7 mg/g), onion (22.15 mg/g), black pepper (0.53 mg/g), pèppè (0.8 mg/g), and parsley (1.4 mg/g) as reported by Oluwatoyin (2014) and Nwinuka et al. (2005). Similar increase in tannins levels was observed with addition of spice in *Nnam gon* formulation. Cake produced from formula 2 had a high amount of tannins (527.44 mg/100 g DM), but low level was observed for control cake (131.59 mg/100 g DM).

### 3.3 Antioxidant properties of *Nnam gon*

**Figure 2:**
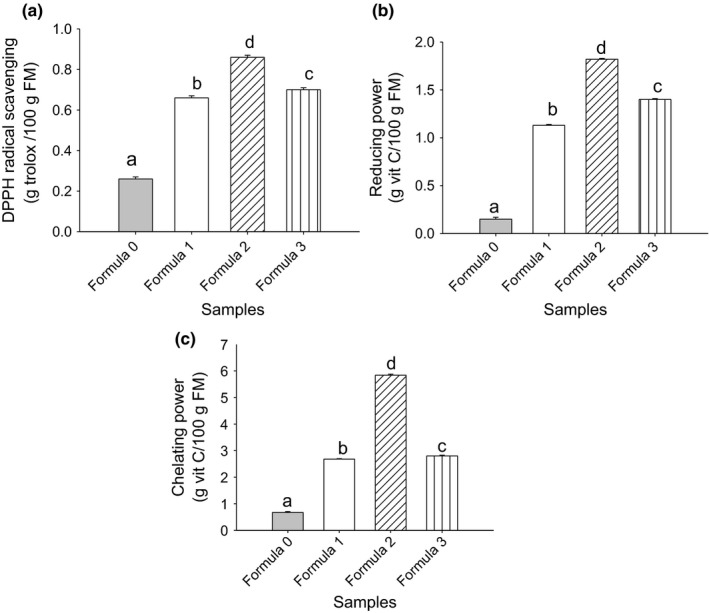
Effect of formulation on DPPH antiradical scavenging (a) reducing power (b) and chelating power (c) of *Nnam gon*. Mean ± *SD* (*n* = 3) followed by different letters is significantly different (*p* < .05) as determined by Duncan's multiple range test

It has already been established that the consumption of foods rich in antioxidants helps to prevent the diseases related to oxidative stress (Boskou, 2006; Saikat, Raja, Sridhar, Reddy, & Biplab, 2010). In general, as shown in Figure 2, the addition of the spices in the *Nnam gon* increased the antioxidant properties of this dish. In fact, antioxidants can provide hydrogen atoms to free radicals and stop the oxidation chain reaction. This mechanism can be illustrated by DPPH Free radical scavenging effect which ranged between 0.26 and 0.86 g Trolox/100 g DM. The reducing power of cakes ranged between 0.15 and 1.82 g Vit C/100 g DM, whereas chelating power was comprised between 0.67 and 5.84 g Vit C/100 g DM. The strong correlations observed between phenolic compounds and DPPH free radical scavenging (*r* = .99; *p* < .05), reducing power (*r* = .99; *p* < .05) and chelating power (*r* = .97; *p* < .05) indicate that these compounds whose antioxidant activity is well established contribute to raising the antioxidant properties of cakes which are enhanced by spices. Moreover, many authors have shown that the spices used in this study exhibited antioxidant properties including onion (Mnayer et al., 2014), garlic (Rahman, Fazlic, & Saad, 2012), ginger (Ghasemzadeh, Jaafar, & Rahmat, 2010), celery (Jung et al., 2011), and parsley (Zhang, Feng, Xi, & Hui‐Yuan, 2006). We should note that the contribution of these spices to increase phytate content of cakes could also induce an increasing in antioxidant properties because of high correlations observed between phytate and DPPH radical scavenging (*r* = .72; *p* < .05), reducing power (*r* = .84; *p* < .05), and chelating power (*r* = .91; *p* < .05). With this regard, some experiments demonstrated antioxidant and anticancer effects of phytic acid in preventive and therapeutic actions (Midorikawa, Murata, Oikawa, Hiraku, & Kawanishi, 2001).

### 3.4 Textural properties of *Nnam gon*

Texture is the most important quality attributes that affect consumer acceptability of cake (Aboua, Konan, Kossa, Agro, & Kamenan, 1989; Aboubacar, Yacizi, & Hamaker, 2006). According to the results presented in Table 4, textural parameters of cakes varied significantly with the formulations. Irrespective of the probe used, Formulation F1 had the lowest textural parameters, hardness and adhesivity were higher in formulation F2 probably due to spices adding. Hardness and adhesivity were positively correlated with protein contents (*r* = .82, *r* = .85; *p* < .05) and total sugar (*r* = .78, *r* = .83; *p* < .05), while viscoelasticity index which represents the property of materials that exhibit both viscous and elastic characteristics when undergoing deformation is higher for the control *Nnam gon* (Formulation F0), but spice seems to reduce viscoelasticity of *Nnam gon* which was highly correlated with phenolic content (*r* = .81; *p* < .05) and phytate (*r* = .85; *p* < .05).

**Table 4:** Effect of formulation on textural parameters of *Nnam gon*

Parameters	Formula/Samples			
	F0	F1	F2	F3
Cylindrical probe				
Hardness (N)	8.46 ± 0.14^b^	5.97 ± 0.13^a^	9.57 ± 0.17^c^	8.60 ± 0.04^b^
Adhesivity (N)	3.19 ± 0.50^a^	4.49 ± 0.32^b^	6.80 ± 0.26^c^	4.75 ± 0.13^b^
Viscoelasticity index	3.12 ± 0.12^c^	1.58 ± 0.19^a^	1.86 ± 0.01^a^	2.26 ± 0.08^b^
Conical probe				
Hardness (N)	8.25 ± 0.03^c^	3.31 ± 0.25^a^	6.74 ± 0.43^b^	8.98 ± 0.02^d^
Adhesivity (N)	5.80 ± 0.02^b^	4.59 ± 0.23^a^	6.69 ± 0.49^c^	8.72 ± 0.37^d^
Viscoelasticity index	2.56 ± 0.18^c^	1.26 ± 0.06^a^	2.24 ± 0.18^b^	2.42 ± 0.16^bc^

Mean ± *SD* values with different letters within the same line differed significantly (*p* < .05) as determined by Duncan's multiple range test (*n* = 03).

### 3.5 Sensory characteristics of *Nnam gon*

The candidates used for sensory analysis were selected based on their ability to discriminate samples. As presented in Table 5, the sensory descriptors of *Nnam gon* samples, developed by the panel, were divided into six main groups: appearance, odor, taste, flavor, texture, and oral texture. Table 6 presents the mean scores for each sample regarding the attributes evaluated. It can be seen that all the *Nnam gon* evaluated have different sensory profiles. Concerning appearance, *Nnam gon* produced from formula 2 showed higher means for color and porosity, whereas those produced from formula 3 exhibited higher means for smooth. In addition, significant correlations were observed between porosity of cake and protein contents (*r* = .86; *p* < .05), lipid contents (*r* = .85; *p* < .05) and total carbohydrate (*r* = .96; *p* < .05), suggesting the strong implication of these molecules on the porosity of *Nnam gon*.

**Table 5:** Sensory describing terms developed for *Nnam gon* samples

Sensory describing terms	Definitions	References
Appearance		
Color	Scale from white to green	0—White paper20—green leave
Porosity	Number of visible pores in the cake	0—shell of the egg20—dry sponge
Smooth	Smooth aspect	0—shell of the egg20—pellicle of pineapple
Odor		
Cucurbitaceae seeds	Sensation of Cucurbitaceae seeds	0—potable water20—Cucurbitaceae seeds flour
Spices	Sensation of spices	0—potable water20—spice powder
Wrapping leaves	Sensation of Wrapping leaves	0—potable water20—Wrapping leaves powder
Fresh fish	Sensation of Fresh fish	0—potable water20—fresh fish paste
Dried shrimps	Sensation of Dried shrimps	0—potable water20—Dried shrimps powder
Musty	Sensation of musty	0—potable water20—moldy bread
Taste		
Cucurbitaceae seeds	Sensation of Cucurbitaceae seeds in the mouth	0—potable water20—cucurbit seeds flour
Persistence of Cucurbitaceae seeds	Taste of Cucurbitaceae seeds that remains in the mouth after rinsing with water	0—potable water20—cucurbit seeds flour
Salty	Intensity of salt	0—potable water20—salt water (0.2 g/ml)
Spices	Sensation of spices	0—potable water20—spice powder
Wrapping leaves	Sensation of Wrapping leaves	0—potable water20—Wrapping leaves powder
Bitter	Bitter taste that remains in the mouth	0—potable water20—fresh aloes vera paste
Bitter after taste	Bitter taste that remains in the mouth after rinsing with water	0—potable water20—fresh aloes vera paste
Flavor		
Cucurbitaceae seeds	Intensity of Cucurbitaceae seeds taste	0—potable water20—cucurbit seeds flour
Dried shrimps	Intensity of Dried shrimps taste	0—potable water20—Dried shrimps powder
Wrapping leaves	Intensity of Wrapping leaves taste	0—potable water20—Wrapping leaves powder
Texture		
Hardness	Strength applied on the cake to break it	0—wheat cake20—cookies
Cohesivity	Facility of cake particles to be separated	0—wheat cake20—cookies
Juiciness	Sensation of humidity on finger	0—bread20—slice of fresh pineapple
Elasticity	Capacity of the cake to return at the start point after a pressure between the fingers	0—cheese20—dry sponge
Oily	Perception of oil between the fingers	0—bread20—cheese
Granulous	Perception of granules between the fingers	0—cookies20—dakere (semolia)
Oral texture		
Juiciness	Humidity in mouth	0—bread20—slice of pineapple
Masticability	Strength applied on the cake to break it in the mouth	0—cheese20—cookies

**Table 6:** Mean quantitative analysis scores for *Nnam gon* samples

	Attributes	Formulation				
		F0	F1	F2	F3	MSD
Appearance	Color	1.50^a^	1.79^a^	2.51^a^	2.31^a^	0.51
	Porosity	6.02^a^	6.99^a^	8.95^b^	7.79^ab^	0.73
	Smooth	10.80^a^	10.10^a^	7.52^a^	8.55^a^	0.71
Odor	Cucurbitaceae seeds	11.98^b^	11.93^b^	9.76^a^	9.88^a^	0.76
	Spices	1.00^a^	5.57^b^	7.38^c^	6.73^bc^	0.88
	Wrapping leaves	5.49^a^	5.51^a^	4.94^a^	4.60^a^	0.85
	Fresh fish	0.00^a^	6.94^b^	5.94^b^	5.49^b^	0.83
	Dried shrimps	0.00^a^	6.16^b^	0.00^a^	5.89^b^	0.79
	Musty	1.87^a^	1.50^a^	1.28^a^	1.41^a^	0.72
Taste	Cucurbitaceae seeds	14.20^b^	10.95^a^	11.36^a^	12.45^a^	0.70
	Persistence of Cucurbitaceae seeds	11.12^a^	11.08^a^	10.17^a^	10.51^a^	0.69
	Salty	8.54^a^	8.55^a^	8.57^a^	8.86^a^	0.59
	Spices	0.00^a^	5.97^ab^	12.18^b^	6.91^a^	0.82
	Wrapping leaves	5.30^a^	5.41^a^	5.99^b^	5.95^b^	0.78
	Bitter	0.00^a^	0.00^a^	0.00^a^	0.00^a^	0.64
	Bitter after taste	0.00^a^	0.00^a^	0.00^a^	0.00^a^	0.64
Flavor	Cucurbitaceae seeds	10.30^a^	9.51^a^	9.35^a^	9.50^a^	0.72
	Dried shrimps	0.00^a^	5.75^b^	0.00^a^	5.76^b^	0.73
	Wrapping leaves	5.25^a^	5.41^a^	5.99^a^	5.95^a^	0.78
Texture	Hardness	10.98^a^	11.77^a^	10.09^a^	10.97^a^	0.69
	Cohesivity	11.02^b^	10.93^b^	8.90^a^	9.31^a^	0.77
	Juiciness	7.69^a^	7.79^a^	8.46^a^	7.61^a^	0.84
	Elasticity	8.92^a^	8.90^a^	8.79^a^	8.88^a^	0.70
	Oily	7.49^a^	7.51^a^	8.10^a^	7.51^a^	0.75
	Granulous	8.05^a^	8.01^a^	7.67^a^	7.67^a^	0.77
Oral texture	Juiciness	7.10^a^	7.02^a^	8.12^a^	7.60^a^	0.82
	Masticability	9.10^b^	9.09^b^	7.94^ab^	6.61^a^	0.66

Means with the same letters on the same line are not significantly different (*p* ≤ .05) and MSD = minimum significant difference (Duncan test of averages).

One of the most important characteristics of good food is its odor. In this respect, general acceptance of *Nnam gon* was highly correlated with spice odor (*r* = .99; *p* < .05). In this study, cake produced with formula 2 and 3 presented the highest means score of spices odor but lower means Cucurbitaceae seeds odor. It could probably be due to the fact that spices odor mask that of Cucurbitaceae seeds odor, hence the negative correlation observed between these attributes (*r* = −.79; *p* < .05). However, formula 1 cake had higher mean score of fish and shrimp's odor, whereas musty odor was observed on control cake F0. Taste and Cucurbitaceae seeds flavor were highly represented in control cake, but addition of spices in cake formulation seems to reduce this attributes. As observed in Table 6, cakes produced with formula 2 and 3 presented the higher mean score of taste of spice. The attributes bitter and bitter after taste were not related for all the samples.

Regarding texture, the sample produced with formula 1 presented the higher mean score of hardness. Spices adding appears to induce a decrease in hardness, cohesivity, elasticity, and granulous of cake, but it seems to enhance the oiliness. This previous observation could mean that spices increase oil absorption capacity of Cucurbitaceae paste. Another parameter that could affect the texture of cake is the moisture content; however, it was not correlated with any texture attribute. Moreover, not only the spicy of paste seems to increase juiciness of cake but it also induced a decrease in cake masticability.

**Figure 3:**
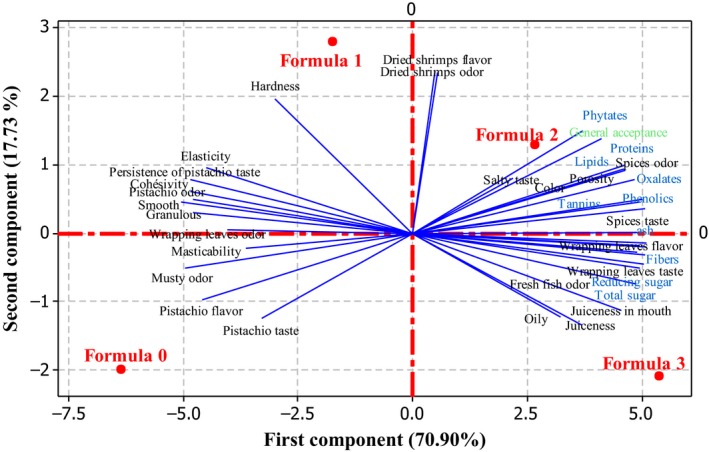
Biplot (CP1 and CP2) describing the mapping of formula and correlations between sensory attributes (black color) and chemical composition (blue color) of *Nnam gon* samples

**Figure 4:**
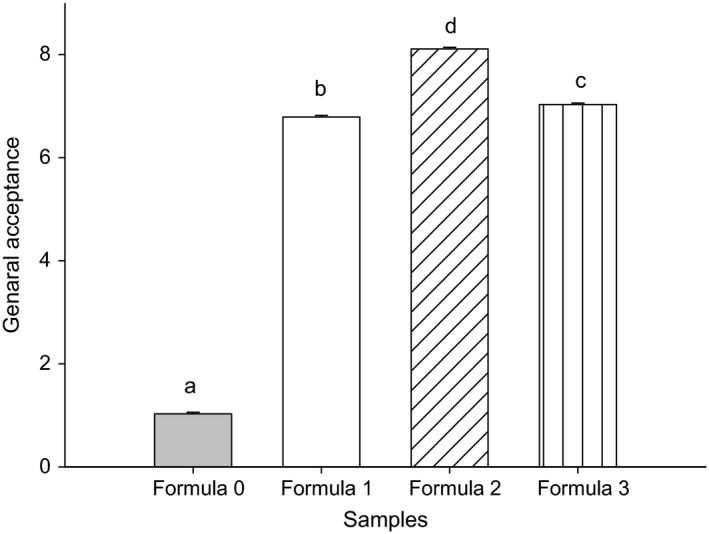
Effect of formulation on general acceptance of *Nnam gon*. Mean ± standard deviation of general acceptance is based on a nine‐point hedonic scale with 1 = dislike extremely to 9 = like extremely. Mean ± *SD* values with different letters differed significantly (*p* < .05) as determined by Duncan's multiple range test (*n* = 50)

In order to study the relations between sensory attribute and chemical composition of the fourth *Nnam gon* samples, the Principal Component Analysis (PCA) was conducted (Figure 3). Principal components 1 and 2 explained, respectively, 70.90 and 17.73% of the variability among the samples. As illustrated in Figure 3, control *Nnam gon* (F0) was characterized by the attributes musty odor, Cucurbitaceae seeds taste and flavor, and persistence of Cucurbitaceae seeds taste, whereas F1 formulated cake was highly correlated with dried shrimps flavor and odor. The attributes that characterized cake produced with formula 2 were color, spice odor, and spice taste. However, formula 3 cake was characterized by fresh fish odor, wrapping leaves taste, and odor. It is interesting to note that the samples were also somewhat separated in terms of spice adding. Then, as seen in Figure 4, cakes produced with spicy Cucurbitaceae paste (formula 2 and 3) had higher mean score for general acceptance which was highly correlated with spice odor (*r* = .99; *p* < .05), spice taste (*r* = .92; *p* < .05), and color (*r* = .84; *p* < .05).

## 4 Conclusion

This study aimed at evaluating the effect of formulation on proximate composition, and textural and sensory properties of *Nnam gon*. The overall differences observed among the samples evaluated are directly related to spices adding in the formulations. Then, the study revealed that proteins, carbohydrate, lipid, ash, and fibers contents of *Nnam gon* increased significantly with spices adding. In addition, the polyphenols and phytates contents of cake increase and are positively correlated with antioxidant properties of cake which also increases with spices adding. The general acceptance of cake is highly correlated with spice odor, spice taste, and color. Cake produced with spicy formulation had higher mean score for general acceptance. However, several species of Cucurbitaceae are edible. Thus, it would be interesting to include this aspect in order to know the species of Cucurbitaceae seeds that are more accepted by consumers for the production of this dish. In this respect, further investigation could be done to determine the influence of species of Cucurbitaceae seeds on proximate composition, and textural and sensory properties of *Nnam gon*.

## Conflict of Interest

None declared.
